# Responses to Vocalizations and Auditory Controls in the Human Newborn Brain

**DOI:** 10.1371/journal.pone.0115162

**Published:** 2014-12-17

**Authors:** Alejandrina Cristia, Yasuyo Minagawa, Emmanuel Dupoux

**Affiliations:** 1 Laboratoire de Sciences Cognitives et Psycholinguistique (ENS, EHESS, CNRS), Département d′Etudes Cognitives, Ecole Normale Supérieure, PSL Research University, Paris, France; 2 Department of Psychology, Faculty of Letters, Keio University, Tokyo, Japan; Kyoto University, Japan

## Abstract

In the adult brain, speech can recruit a brain network that is overlapping with, but not identical to, that involved in perceiving non-linguistic vocalizations. Using the same stimuli that had been presented to human 4-month-olds and adults, as well as adult macaques, we sought to shed light on the cortical networks engaged when human newborns process diverse vocalization types. Near infrared spectroscopy was used to register the response of 40 newborns' perisylvian regions when stimulated with speech, human and macaque emotional vocalizations, as well as auditory controls where the formant structure was destroyed but the long-term spectrum was retained. Left fronto-temporal and parietal regions were significantly activated in the comparison of stimulation versus rest, with unclear selectivity in cortical activation. These results for the newborn brain are qualitatively and quantitatively compared with previous work on newborns, older human infants, adult humans, and adult macaques reported in previous work.

## Introduction

In the adult brain, speech can recruit a brain network that is overlapping with, but not identical to, that involved in perceiving non-linguistic vocalizations [1]. While a body of behavioral evidence suggests that both linguistic and non-linguistic vocalizations are salient even to newborns [2], much less is known about the functional development of the neural structures subtending their perception. The present study sought to document responses to different vocalization types in the human newborn brain.

Vocalizations are of such importance to social animals that primates, whose social structure is complemented by a rich communicative inventory, may have become auditory specialists [3]. It has been proposed that the cerebral mechanisms recruited to perceive and decode vocalizations may be similar in humans and other primates [4]. In view of their evolutionary precedence, one would expect that vocalizations should be processed by structures in the human brain that become functional very early on. Moreover, vocalizations are likely salient experiences in the fetus' auditory world, and by birth neonates have accumulated a certain amount of experience with them. In fact, the mother's linguistic and non-linguistic vocalizations are available to the child through bone and fluid conduction, and the auditory system is mature enough to process them by 3 months before full-term birth [5]. Thus, both evolutionary precedence and *in utero* experience should converge in promoting the early development of the brain networks subtending the perception of vocalizations in newborn humans.

Numerous studies have investigated the neural correlates of speech processing in human infants, including newborns (a recent summary in [6]). Fewer have documented the development of cortical networks processing vocalizations, and particularly emotional ones, and they have thus far focused mainly on infants between 3 and 7 months of age (e.g., [7]). There is even less functional Magnetic Resonance Imaging (fMRI) or functional Near InfraRed Spectroscopy (fNIRS) evidence on infant processing of conspecific compared to non-conspecific vocalizations, with the sole exception of [8] as detailed below.

The latter gap is particularly relevant given some very intriguing results from behavioral preference paradigms, where presentation of sound is contingent on a sucking response from the infant. Such work has recently revealed that human infants exhibit no preference between speech and macaque calls at birth, although they do so by 3 months of age [2]. Moreover, newborns exhibit as robust a preference for monkey calls over synthetic sounds [2] as they do for speech over synthetic sounds [9]. This behavioral research on vocalization processing appears to align with previous work on face perception, which documents perceptual narrowing: Whereas younger infants are equally capable of discriminating individual faces of monkeys as they are of humans, older infants and adults excel only at the latter [10]. Nonetheless, newborns' brain may treat the two stimuli differently in ways that are not evident in the infants' preference behavior. A neuroimaging investigation may be more sensitive to differential neural processing.

At present, only one report has addressed this question. Shultz and colleagues studied infants between 1 and 4 months of age using fMRI [8]. They presented several types of auditory stimuli, including human speech, human emotional vocalizations, human non-communicative vocalizations, rhesus monkey calls, and sounds of walking. Results revealed that several regions in the left temporal and frontal cortices responded to all auditory stimuli, and significantly or marginally more strongly for speech than the other signals. A follow-up analysis suggested that, as infants age, these regions become significantly less responsive to non-speech sounds (including communicative and non-conspecific vocalizations), gaining in selectivity. Thus, although newborns where not tested, these recent fMRI results suggest that the lack of preference between human and non-conspecific vocalizations observed shortly after birth may truly indicate similar processing of the signals at the neural level.

**Figure 1:**
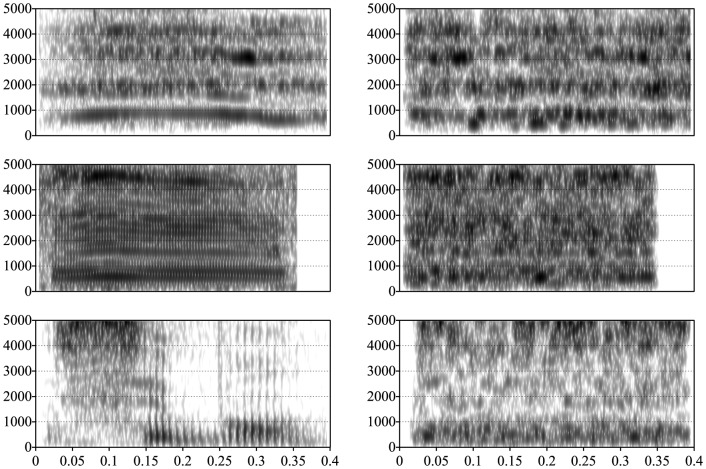
Examples of acoustic characteristics of the stimuli. The top left is an example from the emotion stimuli; the middle left, from monkey vocalizations; and the bottom left, from speech. The three examples on the right are the scrambled counterparts of the corresponding stimulus on the left.

The data presented here directly address this statement, and could provide independent support to Shultz' findings. To shed light on the development of brain networks involved in vocalization processing, we collected neuroimaging data on human newborns using native and foreign speech, human emotional vocalizations, macaque calls, and auditory controls. Similar stimuli have been used in two published studies: Minagawa and colleagues, who reported fNIRS data from 12 Japanese 4-month-olds [11]; and Joly and collaborators, who reported fMRI evidence from 3 adult macaques and 20 French adult humans [12]. We next introduce the 5 types of stimuli (see Stimuli section and Fig. 1 for more detail), and how each of these has been implemented in our and the aforementioned related studies. For ease of expression, we refer to these two datasets using the name of the first author of those published studies.

We have all used a common set of stimuli representing macaque calls and human emotional vocalization. Additionally, both Joly and we had a French speech condition, which mapped onto the human listeners' native language or L1, and to speech more generally for the macaques. We both also included an Arabic speech condition (in Joly's case, only presented to the human participants), which mapped onto a foreign language, or L2. These abstract conditions were also presented to the 4-month-olds in Minagawa's data, albeit implemented in Japanese and English stimuli respectively.

Finally, we included auditory control conditions. Given that the vocalizations used were very short, their key identifying feature was a clear formant structure. Therefore, our auditory control was made by scrambling (see Materials & Methods for further details), which destroys the general formant structure while preserving the long-term spectrum and duration of auditory stimuli. Although backward speech is a common control in previous infant work when the focus is in fine-grained temporal properties (e.g., [13]), it was not appropriate here, as the focus was in formant structure and not temporal features. Whereas adult participants in Joly et al. heard the four types of scrambled stimuli in separated trials, the infant participants in both Minagawa-Kawai's study and ours heard only a subset of them, and no separate analyses were performed.

**Figure 2:**
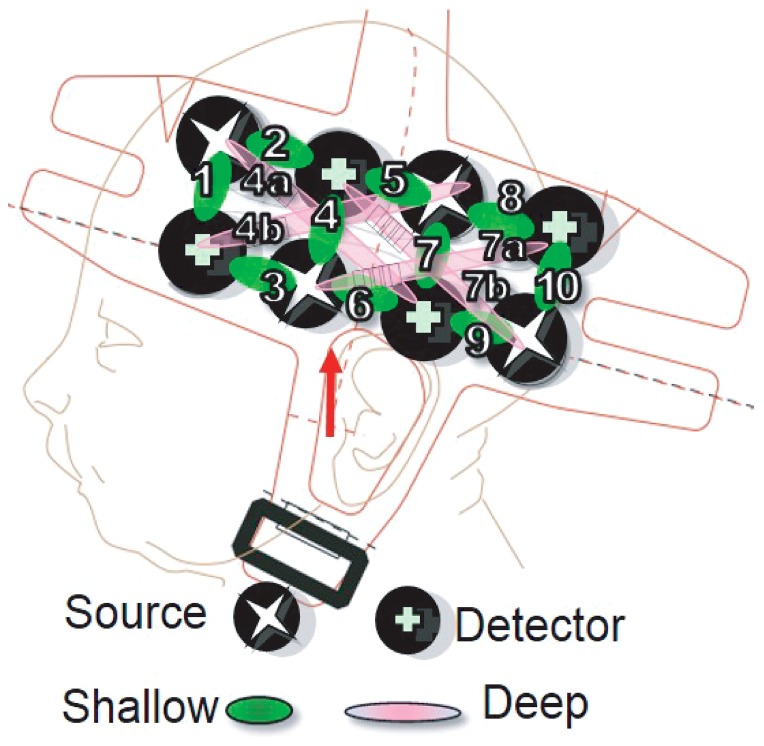
Location of the 10 shallow channels (1–10) and 4 deep ones (4a,b and 7a,b) on a model of a newborn's head. See Fig. 4 for an estimation of the point of maximal sensitivity for each channel.

By using the same stimuli as previous work [11], [12], we can directly address the question of whether sensitivity and selectivity for vocalizations changes with development. Responses were estimated using fNIRS measurements gathered from bilateral perisylvian cortices (see Fig. 2). Based on the results of this previous work (see Results section), and if the null hypothesis is false, one would expect to find greater activation for native than foreign speech; and for speech than for macaque vocalizations in the cortical regions sampled by our probes. Indeed, the human adult results revealed greater activations for native speech than non-native speech in both temporal and frontal cortices. Only temporal cortices were measured at 4 months, when greater activation for native than non-native speech was evident in temporal cortices bilaterally. Additionally, the human adult data revealed greater activations for human speech than emotion and macaque vocalizations in regions including the temporal cortices whereas no difference was found in this contrast in the adult macaque data. (These contrasts were not reported on in the 4-month-old study.) The null hypothesis against which this prediction is tested is, therefore, that there will be no significant difference in the following contrasts: native versus scrambled control; native versus foreign speech; native versus emotional vocalizations; native versus macaque vocalizations. In addition to comparing our results with those obtained with the same stimuli presented to different populations, we also integrate our findings with other infant neuroimaging work.

## Results

**Figure 3:**
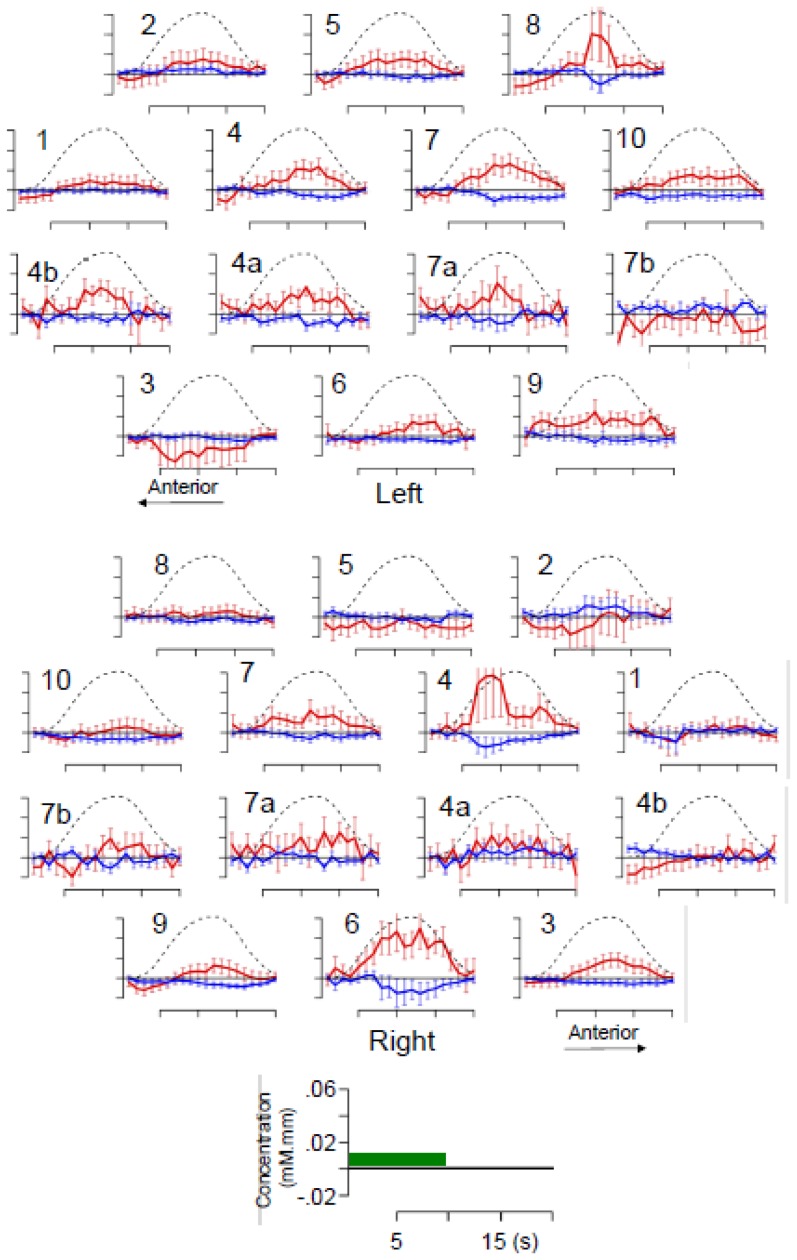
Average time course of the hemoglobin changes following auditory stimulation in individual channels revealed by a general linear model. The trace in red represents oxyHb, in blue deoxyHb; the error bars indicate standard error (over participants). The zero level or baseline is defined as the intercept of the linear model. The black dotted lines show the standard HRF model convolved with the average duration of stimulation, scaled to the maximum average concentration. The scale as well as the timing of stimulation (green box) are shown in the reference axes.

**Figure 4:**
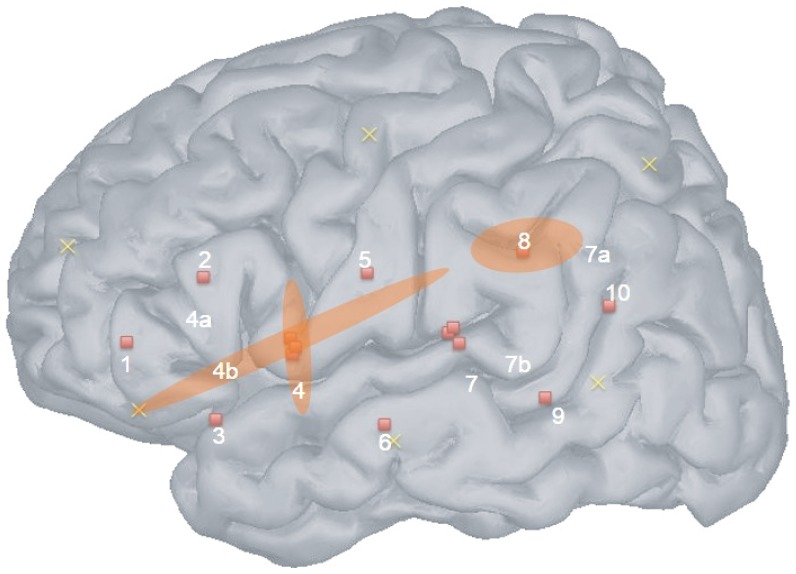
Channels responding significantly to auditory stimulation. The three channels indicated had significant activation in the analysis where all stimulation was declared together against the silent baseline.

The GLM declaring all conditions together against the silent baseline highlighted three channels, all of them in the left hemisphere (see Fig. 3, and Table 1). One of them plausibly travels through superior temporal gyrus, inferior frontal gyrus, and precentral cortices (shallow channel 4), another traverses the entire superior bank of the perisylvian cortices (thus covering some frontal, precentral, and postcentral cortices; deep channel 4b), and a third is more posterior, a channel estimated to traverse the angular and supramarginal gyri in addition to posterior temporal regions (see Fig. 4). The ANOVAs on the sound-sensitive channels revealed significant intercepts [channel 4: F(1,27)  =  5.7; channel 4b: intercept F(1,27)  =  4.7; channel 8: intercept F(1,29)  =  5.2], and no significant main effects or interactions [in channel 4, other Fs <3.1; channel 4b, Fs <2; channel 8, Fs <1.7]. Thus, although the presence of sounds was detected (the intercept indicates significant changes in concentration during stimulation compared to baseline), no significant difference across conditions was observed in these data.

**Table 1:** Channels activated in the sound versus silence contrast.

All conditions versus silence													
Left							Right						
Ch	*β*	SE	t	N	p unc	p cor	Ch	*β*	SE	t	N	p unc	p cor
1	0.01	0.004	2.25	39	0.031	0.41	3	0.013	0.005	2.73	37	0.01	0.16
4 *	0.017	0.005	3.18	39	0.003	0.05	6	0.02	0.008	2.63	38	0.012	0.19
5	0.013	0.005	2.45	39	0.019	0.29	7	0.012	0.005	2.51	40	0.017	0.25
7	0.018	0.006	2.76	40	0.009	0.15	9	0.013	0.005	2.38	39	0.022	0.33
8 *	0.018	0.005	3.53	39	0.001	0.02							
10	0.011	0.004	2.58	36	0.014	0.22							
4b *	0.016	0.004	3.55	39	0.001	0.02							

Ch  =  channel (channels with a number followed by a subscript are deep); *β* =  beta recovered from the GLM in mM.mm; SE *β*  =  standard error of the *β*, N  =  number of children contributing data for that channel and condition, t value, p unc(orrected), and p cor(rected through resampling). Only channels whose *β* value was significantly different from zero at p ≤ 0.05 uncorrected, for the relevant condition, are shown. Channels with significant *β* after correction through resampling are marked with *.

When each condition was declared separately, several channels were activated at uncorrected levels, as evident from Table 2.

**Table 2:** Channels activated in the five conditions.

Left							Right						
Ch	*β*	SE	t	N	p unc	p cor	Ch	*β*	SE	t	N	p unc	p cor
Macaque calls													
5	0.022	0.01	2.17	33	0.03	0.54							
4b	0.016	0.008	2.09	32	0.05	0.61							
Human emotional vocalizations													
2	0.03	0.011	2.7	34	0.01	0.22							
4	0.031	0.01	3.03	32	0	0.11							
8	0.02	0.01	2.02	33	0.05	0.69							
Native speech													
7	0.022	0.009	2.37	34	0.02	0.38	7b	0.025	0.009	2.65	33	0.013	0.23
Foreign speech													
7	0.025	0.012	2	34	0.05	0.71	4a	0.025	0.008	2.93	31	0.006	0.13
Scrambled auditory control													
4a	0.023	0.009	2.64	34	0.01	0.23	7	0.031	0.013	2.4	33	0.022	0.35
							7a	0.032	0.011	2.93	32	0.006	0.12

Ch  =  channel (channels with a number followed by a subscript are deep); *β*  =  beta recovered from the GLM in mM.mm; SE *β*  =  standard error of the *β*, N  =  number of children contributing data for that channel and condition, t value, p unc(orrected), and p cor(rected through resampling). Only channels whose *β* value was significantly different from zero at p ≤ 0.05 uncorrected, for the relevant condition, are shown.

The results from direct contrasts across conditions are conveyed in Table 3, composed to facilitate the comparison with the other populations tested with the same stimuli.

**Table 3:** Comparison between the newborn results reported here, and published results from human 4-month-olds [11], adult humans and adult macaques [12].

	Newborns	4-month-olds	Adults	Macaques
Sound vs. Silence	* L4 L8 L4b	* Lc Rc		
Scrambled vs. Silence	# L4a R7 R7a	# Lc	* LS STG STS	* STG STS F
Macaque vs. Silence	# L5 L4b	* L and R b-d		
Human Emotion vs. Silence	# L2 L4 L8	# Rc		
Native vs. Silence	# L7 R7b	* La Lc Ld		
Foreign vs. Silence	# L7 R4a	# Lc Ld		
Native vs. Foreign	None	Lc Rc	* STS, IFG	(–)
Human Emotion vs. Macaque	# -R3		# STS	None
Native vs. Human Emotion	# -L2 R1 R7b		# STS STG IFG	None
Native vs. Macaque	# R7b		# STG STS IFG	None
Foreign vs. Human Emotion	None		* STG STS	(–)
Foreign vs. Macaque	None		* STG STS	(–)

The symbol indicates significance level with * at a corrected level (through resampling in the present work, FDR in the 4-month-old study, and FWE in the adult work) and # at an uncorrected level (.05 for the infant work,.005 for the adult work); ‘none’ indicates None at p<.05 uncorrected for the infants, and at p<.001 for the macaques. The Native vs. Foreign contrast in the 4-month-olds was significant at uncorrected p  = .05 in an ANOVA including both channels. A negative sign indicates that a difference was counter to the stated order (e.g., more activation for macaque calls than human emotional sounds in the newborns). Empty cells were not reported; those with (–) involve stimuli not presented to that population. The channels in [11] have been renamed a through c here for ease of reference. LS  =  lateral sulcus, STS  =  superior temporal sulcus, STG  =  superior temporal gyrus, F = frontal regions, IFG  =  inferior frontal gyrus. All activations in adult monkeys and humans were present in both hemispheres, with various degrees of dominance (not represented here).

In sum, based on these results, we have failed to rule out our null hypothesis. The pattern of results noted above was stable across a reasonable variety of analysis parameter choices, including one that matched exactly those used in the 4-month-old study and another with a stricter threshold for artifact determination (see Supplementary Analyses on https://osf.io/q2mxe/files/for tables showing the results of the present and the alternative analyses).

### Comparison with previous studies using the same set of stimuli

One of the motivations for the present study was to facilitate a comparison with other populations presented with the same stimuli, namely the 4-month-old Japanese infants reported by Minagawa in [11] and the human French speakers and adult macaques reported by Joly in [12]. For this comparison to be meaningful, it is important to ensure that all datasets have been gathered under comparable conditions.

**Figure 5:**
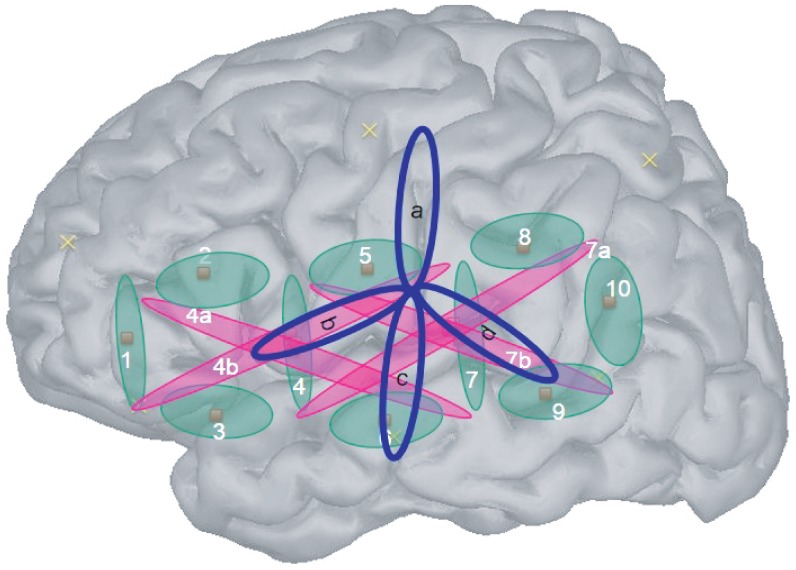
Approximate localization of channels in the 4-month-old study (in blue) overlaid over our shallow (in green) and deep (in pink) channels.

Overall, the infant and newborn studies are quite similar, except for some salient features: The fNIRS system (same wavelengths, but different machine and different probes - though see Fig. 5 for similarity in coverage); the stimuli for L1 and L2; and the fact that infants were awake whereas many newborns were asleep. Other than that, the exact same stimulation paradigm has been used between the newborn and 4-month-old results.

The divergence is greater with respect to the adult studies, which were much longer, had several scrambled stimuli that matched the separate conditions, and were collected using fMRI. In the case of the adult monkeys, an agent was injected to increase contrast-to-noise ratio. Moreover, it is important to bear in mind that many across-condition comparisons in the adult work use a double subtraction; for example, the Native versus Foreign contrast is actually (Native minus Native-Scrambled) minus (Foreign minus Foreign-Scrambled). Particularly in view of these divergences, how can we ensure that results can be meaningfully compared across such diverse settings? One first concern relates to tissue sampling, which is more precise in fMRI than fNIRS in general. Neither of the pads used with infants sampled densely, and while source-detector pairs at 25mm may sample from all tissue beneath, sensitivity in the the sampled volume is lower in the ‘edges’ than in the ‘center’. Moreover, the regions that were most saliently involved in contrasts among adults were localized to the STS, which may not be easy for fNIRS to tap, and the STG, which was variably covered by the two fNIRS studies. Nonetheless, given the extensiveness of the speech-selective regions uncovered by Shultz and colleagues [8], it appears unlikely that we would have failed to tap the same regions that are selective in slightly older infants.

Setting aside this sampling concern, are signal-to-noise ratios comparable across human newborns, 4-month-olds, and adults, as well as the adult macaques? Joly and collaborators also faced a comparability problem across the two populations they investigated. In that paper [12], the authors argued that power was matched because areas of similar extent were activated at a similar corrected level when data from the two populations were investigated in the scrambled versus silence contrast.

Using the same criterion, we must conclude that the 4-month-old study had less power than the adult work. This is not surprising, as the adults heard many more trials (particularly of the scrambled type) than these older infants did. Moreover, by the same token, the newborn study had even less power than the 4-month-olds, as no channel was activated for the scrambled trials even at a lax criterion. Such a decrease in signal-to-noise ratios cannot explained by a difference in stimulation, as the exact same presentation protocol was used and almost precisely the same stimuli were used in neonates and 4-month-olds. This is also not due to an overall much poorer fit, amongst newborns, of the standard HRF model used for the GLM analyses. For the 4-month-olds the median R oxyHb was.99, versus.96 for the newborn shallow channels.

Based on these indirect comparisons, we conclude that signals were somewhat stronger in the infant than the newborn study, and a good deal stronger in the human and macaque adults' case than the two fNIRS studies. We return to this in the Discussion.

### Comparison with previous newborn fNIRS research

We found significant overall auditory activations in three channels, but little evidence for activations specific to particular stimulus types. A priori, it is difficult to decide whether this is evidence for weakness of activations (low sensitivity) and/or little differentiation in neural responses across stimuli type (low selectivity). To have a better grasp of which of these descriptions applies to our results, we compared some effect sizes found here against those reported in similar previous newborn fNIRS work.

Comparable previous literature was identified through searches in the online database DBIfNIRS [14]: 9 fNIRS experiments have investigated the newborn brain's response to speech or other vocal signals, with pads positioned over similar regions (likely tapping perisylvian cortex). Most of them have been reported in journal articles [13], [15]–[20] with only one coming from an unpublished thesis [21]. To this sample, we added a previous experiment of ours that did not use linguistic stimuli. In this other experiment, we investigated the modulation of brain activation depending on whether sequences of simple tones with fast versus slow rates of changes were presented [22]. It is relevant here because the data for that study were collected from the same newborn population using the exact same equipment used here (in fact, they were collected roughly at the same time and by the same experimenters), and analysed using the same procedures. Further information on how effect sizes were calculated can be found in the online supplementary materials, https://osf.io/q2mxe/files/.

We first investigated potential differences in power as well as the stimuli presentation procedure, shown in Table 4. This table clearly shows that our study does not suffer from a small sample size: with between 31 and 39 infants for any given comparison, it actually has the largest sample size. It also does not have overly few trials, although much information is missing on this count. Our study is average in terms of total number of data points, estimated as participants times maximum trials. Notice that this calculation does not take into account attrition, but our study (as reported in section 2.4.1) fares well on that count.

**Table 4:** Comparison of amount of data between the newborn results reported here, and previous comparable infant work.

First author	Sample size	Min trial N	Max trial N	Max Tot
Shultz*	24	2	5	120
Arimitsu	17	7	7	119
Gervain (exp. 1)	22		14	308
Gervain (exp. 2)	22		14	308
Kotilahti	13		64	832
May	20		7	140
Nishida	10	5	10	100
Peña	14		10	140
Sato	17	2	6	102
Shukla	25	10	30	750
Minagawa	28	10	24	672
Present study	39	4	8	312

Min/Max trial N stands for the minimum and maximum number of stimulation blocks or trials. Max Tot is calculated by multiplying the sample size by the maximum number of trials. White cells indicate unavailable information. Except for Shultz*, all studies focused on newborns and used fNIRS (see main text for details).

Next, we sought to estimate the size of the effects reported in such work. When multiple effect sizes could be calculated from the same study (e.g., multiple channels or regions of interest reported), we selected the channel or group of channels that had given the lowest p-value or the highest t-value. Notice that this decision over-estimates the size of the effects found in previous literature. Effect sizes could thus be calculated from reported means and SD for two experiments and our previous data, from exact t-values in 2 additional cases, and from exact (uncorrected) p-values for two more. When multiple contrasts were reported, we prioritized contrasts between conditions (possible in 3 cases, shown in Table 5), which spoke to the question of selectivity, over comparisons between stimulation and silence (the remaining 4 cases, shown in Table 6), which speak to the question of sensitivity or strength of activation. We also compare the present data against our previous newborn data in both tables.

**Table 5:** Comparison between the newborn results reported here, and previous newborn selectivity results (i.e., dissimilarity in strength of responses across conditions).

First author	Condition 1	Condition 2	Localization	Effect Size
Arimitsu	phonemic change	no phonemic change	left temporal	1.323
Gervain (exp. 1)	ABB (syllables)	ABC (syllables)	left temporal	0.814
Shukla	full sentences	backwards sentences	left temporal	0.575
Minagawa	fast-changing tones	slow-changing tones	right perisylvian	0.582
Present study	brief phrases	scrambled	right perisylvian	−0.344

Localization and channel number provide information on the one channel whose data was used for the effect size. In all cases, the effect size is Cohen's d indicating selectivity of responses, in the sense of greater reaction to Condition 1 than Condition 2. For comparability with the previous research, the effect size for our study comes from the native speech versus scrambled controls. Please see main text for details on how effect sizes were selected and calculated.

**Table 6:** Comparison between the newborn results reported here, and previous newborn speech sensitivity results (i.e., strength of responses).

First author	Stimulation	Localization	Channel number	Effect Size
Kotilahti	full sentences	left	max in 8	1.184
May	LPF full sentences	left	average of 6	0.242
Nishida	full sentences	left perisylvian	average of 7	1.153
Minagawa	fast-changing tones	left temporal	1	0.979
Present study	brief phrases	left temporal	1	0.419

Localization and channel number provide information on the channel(s) whose data was used for the effect size. In all cases, the effect size is Cohen's d indicating the size of the response to speech against a baseline of silence. LPF stands for low-pass filtered. Please see main text for details on how effect sizes were selected and calculated.

To represent the present data, we selected the most responding channel in the contrast of native speech against silence, and native speech against scrambled auditory stimuli. The latter yielded a negative effect size because the response in a right-hemispheric channel was greater for the scrambled stimuli than for speech. Results of this investigation are shown in Tables 5 and 6.

Table 5 shows that selectivity was more pronounced for the other studies than for current data, meaning that differences across conditions were less reliable here. To a certain extent, this might be due to weak activations found in the present data: Table 6 suggests that, in general, stronger responses have been found for speech in previous work than in the present study, meaning that the native speech used here elicited smaller and/or more variable activations. Therefore, we conclude that the French newborns tested with the present stimuli exhibited both weaker responses and less selective ones than newborns tested in previous work with other speech stimuli. In fact, the responses observed here were weaker than those of neonates exposed to non-linguistic auditory stimuli (simple tones, with faster and slower rates of presentation), but who had otherwise been tested in the exact same conditions (drawn from the same population, tested in the same setting, with the same equipment, and by the same experimenters; and whose data had been analyzed with the exact same procedures and parameters used in the present experiment). In the Discussion, we will explain why responses have been weaker and less selective in the present work than in previous newborn fNIRS research.

## Discussion

The present study sought to document how the newborn brain responds to auditory stimuli as a function of whether they are speech, emotional signals, or non-conspecific vocalizations. We observe that fronto-temporal and parietal cortices respond to auditory stimulation in general. Significant responses were located exclusively in the left hemisphere. Other work has documented significant responses to auditory stimulation, including speech, also in the right hemisphere (to give two examples, [15], [17]). The inconsistency between our results and those ones might be explained as follows. Our stimuli were very short and variable, and thus did not have a clear intonational/prosodic and spectral content that engaged the right hemisphere to any great extent. Notice that all previous work finding strong responses in the right hemisphere in newborns used stimuli with rich and salient prosodic patterns. Naturally, our stimuli being sound, primary right-hemispheric auditory cortices must have been activated. That we fail to find channels in the right that survive correction may indicate that whatever activations are present are too small, too variable across infants, and/or too deep to register with our equipment. In any case, what we do find is that when we declare the contrast between presence versus absence of auditory stimulation, three left-hemispheric channels are found to be significantly activated.

Secondly, we have found no evidence of selectivity in localization or degree of activation. Additionally, we have documented that the newborn responses were weaker than those found in older infants and adults tested with the same stimuli. We further bolstered this finding through a comparison with previous newborn fNIRS research, which had yielded stronger and more selective responses to speech. We now discuss how these findings connect with previous literature in two ways. First, we integrate them with related work using the same stimuli and/or assessing the same question. Second, we discuss some experimental features that may have led to weaker and less selective responses.

### Developmental changes in vocalization processing

We document weaker and non-selective responses to vocalizations in human newborn perisylvian regions, thus providing independent evidence to the conclusion extrapolated from recent fMRI work with slightly older infants [8] that selectivity in responses to speech and conspecific vocalizations is low at birth.

Moreover, since we employed the exact same auditory stimuli as two previous reports on older infants and adults, we can replicate and extend Shultz et al's findings regarding changes with age [8]. Specifically, using a contrast of greater activation for speech over non-speech biological signals, they identified several potentially selective regions in the temporal cortex spanning from the superior to the inferior gyri, and from the posterior temporoccipital cortices to the temporal pole. It was in these regions that a significant decline in responsiveness to non-biological signals was observed, which contrasted with a slight (non-significant) increase in response to speech, as a function of infant age between 1 and 4 months. Given the extensiveness of this area, it should in principle be possible for us to assess whether the conjunction of the present data with Minagawa's data (reported in [11]) conceptually replicates a developmental trend for stronger selectivity with age.

Close inspection of the 4-month-old results reported by Minagawa in [11] suggests that the responses recorded in that study were only weakly consistent with selectivity. Although a direct contrast between the two speech conditions and the non-speech vocalizations were not reported, given the close overlap in terms of channels, we could derive a contrast statistic from the betas and SD reported in that article. This analysis failed to reveal any significant difference, even at p.05 uncorrected, for any of the other contrasts. Thus, neither the newborns nor the 4-month-olds display selective responses to the different stimuli types employed in the present line of research.

These infant results stand in stark contrast with those from the adult humans tested with the exact same stimuli, who exhibited differential levels of activation for every one of the following contrasts: L1 versus L2; speech versus non-speech vocalizations; and conspecific versus non-conspecific. Thus, every dichotomy that is thought to be important was reflected in differences in activation strength, and sometimes activation localization, in adult data. Notice, however, that the human adult patterns differs from that found among the adult macaques, who failed to show a grading in response strength across comparable types (macaque vocalizations compared to human linguistic and non-linguistic ones), even at a very lax correction criterion.

Therefore, two salient possibilities suggest themselves. First, it is possible that the salient selectivity evident in superior temporal cortices found in human adults with the same stimuli is partially function of their unique life experience. For example, one could hypothesize that the production of speech and emotional signals may play a key role in wiring human brain networks such that they come to respond in a highly specific manner.

Shultz and colleagues' results (reported in [8]) suggest that a second hypothesis should also be entertained, and may have contributed to the developmental changes documented through the present work. Indeed, given that these authors have uncovered some selectivity in responses using a different set of stimuli, we must allow for the possibility that the stimuli used in the present study, or its presentation procedure, may not have been ideal to allow such selectivity to surface. Indeed, as noted repeatedly, the responses we measured were weaker than both those found in other populations tested with the same stimuli, and in the same population (newborns) tested with different stimuli. At this point, we turn to the second part of the discussion, where we put forward likely explanations for the weak and non-selective responses observed in the present study.

### Study features leading to weaker and non-selective responses in newborns

We would like to argue that the most compelling explanation for the weaker and non-selective responses observed here relates to the stimuli and presentation procedure. We bolster this claim through a methodological comparison with previous relevant research, summarized in Table 7.

**Table 7:** Comparison of procedure between the newborn results reported here, and previous comparable infant work.

First author	Duration	Min rest	Max rest
Shultz*	20	12	
Arimitsu	15	15	
Gervain (exp. 1)	18	25	35
Gervain (exp. 2)	18	25	35
Kotilahti	5	15	
May	19	25	35
Nishida	15		
Pea	15	25	35
Sato	10	20	30
Shukla	15	25	35
Present study	10	8	16

Duration is the average stimulation duration. Min and Max rest indicate the minimum and maximum duration of the silence following a block. When only the minimum rest duration is noted, only average and not the range was reported. The other white cells indicate unavailable information. Except for Shultz*, all studies focused on newborns and used fNIRS (see main text for details).

Table 7 shows that our study differs from previous ones in terms of stimuli presentation procedure. In our study, a train of stimuli lasted about 10 s, whereas stimulation blocks in previous work tend to be longer, with the median being at 15 seconds. We left only about 12 s of silence to allow a return to baseline, while in most studies this rest duration oscillated between 25 and 35 s. We hasten to explain that our use of GLM allows such a fast presentation rate because it can separate the contributions to a hemodynamic response from different stimuli types. This is unlike methods based on averaging, where the silence between two blocks must be long enough to allow a complete return to baseline of the response to the previous stimuli. Thus, our use of GLM makes it unnecessary to have long rest periods to be able to statistically separate the effects of the different stimuli types in our experiment. However, as we will explain below, using short silences may have impaired *infants'* perception of each type, possibly by loading their memory.

There is a second aspect of our stimuli that may have made the perceptual task challenging. As noted in the Methods section, our stimuli were very well matched to each other, short, and highly variable. Indeed, most of the individual stimuli (i.e., a sentence, call, cry, etc.) used here were selected to be relatively simple, thus blurring some of the differences between stimuli type and potentially preventing their rapid identification. Moreover, each individual stimulus was acoustically different from all others within the same type (e.g., many different voices were used in the speech conditions), and they were all shorter than 1 s in duration. This is very different from most previous work demonstrating stronger and more selective responses to the native language (see Tables 5 and 6): Nearly all that work employed full, potentially complex, infant-directed sentences, spoken by only one talker per language (the only exceptions are [15], [16], who used one bisyllabic word and trains of three monosyllabic words, respectively). When the duration of individual sentences is reported, each is about 3 to 4 s long (e.g., [18]).

All of these methodological parameters are likely to affect infants' discrimination. Indeed, in previous work we have found that increasing the duration of stimulation and decreasing variability boosts discriminability even in adults [23]. Furthermore, behavioral research has documented that neonates' discrimination between rhythmically dissimilar languages can break down when multiple different voices are used to represent each language [24], [25].

In other words, by employing the exact same stimuli as Joly's work on adult humans and macaques [12], task difficulty was not equated across ages, and rendered comparison with other infant work more difficult, as our newborns may have been less able to identify, on the fly, the category differences rendered subtle by using short, variable, and carefully matched stimuli, presented at a fast rate. More successful comparisons across diverse ages may thus necessitate adaptation of the stimuli to each stage in development by slowing presentation rate, rendering the stimuli longer in duration, and possibly reducing the within-type variability to facilitate their identification. Additionally, comparisons across studies within the same age may gain through the standardization of presentation procedures.

### Conclusions

The present study sought to inform the research on the neural bases of vocalization processing in primates. It is the first report on the human newborn brain's responses to native and foreign speech, emotional human vocalizations, macaque calls, and auditory controls. We uncovered weak and non-selective responses to the different types. This result contrasts both with previous reports on newborns using different stimuli but the same technique, and with previous reports on human adults using a different neuroimaging method but the same stimuli. We have argued that our results are likely due to both newborns' being less skilled at identifying the stimuli types with somewhat impoverished stimuli and fast presentation rates, in addition to, possibly, a lower degree of differentiation in cortical responses. We hope future work will continue to investigate the fascinating question of how selective brain networks for vocalizations emerge in the human brain. The present report should facilitate those discoveries by underlining the methodological features that promote strong and selective responses to auditory stimuli in this population.

## Materials and Methods

### Participants

Caregivers of all participating neonates signed an informed consent form. This research project was approved by the Ile-de-France III Ethics Board [No. ID RCB (AFSSAPS) 2007-A01142-51]. Forty newborns (19 girls, 21 boys) were tested between 0 and 6 days after birth (M  =  2, SD  =  1.2) and included for analysis. An additional 3 did not start the study due to being overly restless, and 6 started the study but could not be included because they had less than 2 minutes of data, or were fussing or restless during the first 5 minutes (after which the experimenter discontinued the study). The neonates' quiet awake versus sleeping state was not consistently coded, and therefore analyses separating the two are not possible. The precise number of datapoints varies across analyses, since we only included the data for an individual newborn's channel if there were at least four unartifacted blocks for each of the relevant conditions.

### Stimuli and presentation procedure

Newborns were presented with 5 different types of stimuli: native speech (French); non-native speech (Arabic); human emotional vocalizations; macaque calls; and scrambled stimuli. In all cases stimulation blocks were constituted of trains of individual stimuli. Each individual stimulus was between 400 and 1200 ms in length in all cases. A block (or train of stimuli) contained about 9–12 stimuli of the same type, with an inter-stimulus interval of 200 ms, for a total of about 10 s in average. The jitter between 2 blocks of stimulation varied pseudo-continuously between 8 and 16 seconds. Twenty blocks (including 4 trials of each condition, which alternated in several pseudo-random orders) were considered a session, which had about 7 minutes in duration, and a maximum of 3 sessions were presented consecutively without stopping (except if the newborn showed signs of discomfort). About 20% of newborns completed only one session, 65% completed two, and only 15% completed all three sessions.

The speech stimuli consisted of sentences, recorded from film dialogues or in the lab, and matched in duration to the emotional stimuli and macaque calls. As a result, they were very short phrases. For instance, one trial contained the following phrases: “pas evident?” *hard?* (560 ms; 3 syllables), “sans aucun dout” *undoubtedly* (850 ms; 4 syllables), “qui a eu cette idée?” *who had this idea?* (985 ms; 6 syllables), “comment allez vous?” *how are you?* (650 ms; 5 syllables in this pronunciation), “C′est bon” *it's OK* (420 ms; 2 syllables), “ils font du bruit?” *do they make noise?* (670 ms; 4 syllables).

The emotional human vocalizations, extracted from the same sources as the speech stimuli, had no linguistic content, and included both positive (e.g., laughs) and negative (e.g., sobs) valence.

Monkey calls were selected on the basis of similar valence to the human emotional sounds (e.g. calls with a positive valence, such as coos, and calls with a negative valence, such as screams). They were drawn from the sound library compiled by Marc D. Hauser (recorded in Island of Cayo Santiago, PR, USA). These short segments were concatenated in the same manner as the human sounds.

Scrambled stimuli were generated by concatenations of the individual stimuli once they had been passed through a gammatone filter bank with 64 channels and processed as follows. In each channel, the signal was windowed with overlapping Hanning windows 25 ms in duration. The windows were then shuffled randomly within a channel and displaced within a time range of 500 ms around its original temporal position. As a result of this process, controls are unintelligible, matched in amplitude, duration, and long-term power spectrum to the original files.

Examples of emotion, monkey, and language stimuli, and their control counterparts, are shown on Fig. 1. This Figure illustrates the effects of window shuffling: If one observes closely the sample emotion stimulus, one discerns clearly a long harmonic centered at around 900 Hz, and a zero right above it. Looking over to its counterpart, it is clear that the amount of energy at about 900 Hz compared to 1200 Hz is preserved across the two, but clearly there is no single recognizable harmonic. Additionally, the figure underlines the similarities and differences across stimuli types. Notice the presence of formant structure in all of the vocal stimuli, which is clearly absent from the controls. It is clear from this that human emotional vocalizations and monkey calls are much simpler in terms of longer stable stretches than human speech. One aspect that is not illustrated here but is also an important difference between the conditions is that both human emotional vocalizations and monkey calls are more repetitive than human speech (for example, both barks and laughter are composed of a harmonic complex repeated 3-10 times; nothing like that can be found in the normal adult-directed speech of the type used in the present experiment).

### Equipment and data acquisition

Stimuli were presented from a loudspeaker at about 75 dB measured at the approximate location of the newborn's head. The neonates were inside their cots while they listened to the stimuli.

Changes in hemoglobin concentrations and oxygenation levels in the bilateral temporal and frontal areas were estimated using fNIRS. Our fNIRS system (UCL-NTS, Department of Medical Physics and Bioengineering, UCL, London, UK, [26]) continuously emits near-infrared light of two wavelengths (670 and 850 nm; with a mean power of 2 mW for each diode). Each source signal is frequency modulated at frequencies ranging between 2 kHz and 4 kHz. The light is picked up by 16 detectors, which sample the received light at 20 kHz and digitize it at 16 bits, and perform a Fast Fourier Transform in order to separate the different source signals, resulting in a 10 Hz reconstructed signal for each source.

We used two pads each containing a 2 × 4 array of optical probes separated by 25 mm (see Fig. 2). This configuration provides data from 10 channels between adjacent source-detector pairs, at a distance of 25 mm; and 4 channels between non-adjacent pairs, at a distance of 56 mm. Such multi-distance results are increasingly common in infant fNIRS research (for instance, [7], [17], [27]). Since the light goes in deeper for the latter, we will call these 4 ‘deep’ and the other 10 ‘shallow’. In these cases, the volumes whose level of oxygenation is being estimated are very likely partially overlapping across adjacent shallow and deep channels, and thus cannot be viewed as either interrogating the exact same volume nor completely independent volumes. This has been discussed to a greater extent in previous fNIRS studies using a similar UCL-NTS system [7], [27]. Channel numbering goes from anterior to posterior, with deep channels coded with the same number as a shallow counterpart that travels in the same region and a letter (a/b).

When placing the cap, the bottom of the pad was aligned with the T3-T5 line using the anatomical landmarks of the 10/20 system. The average circumference was 35 cm (minimum 31.5- maximum 38); the average nasion-inion distance was 23 cm (20–27); the average ear-to-ear over vertex distance was 23 cm (21–26). Localization estimations were drawn based on a set of published probabilistic maps aligning 10–20 system landmarks to underlying anatomy [28]. To use these published maps, we homothetically scaled these adult-based landmarks to the head size of an average newborn, and estimated the point of maximal sensitivity for each channel using virtual registration. We took into account two types of measurement error: the estimates of positions in the probabilistic maps used have an SD of approximately 8mm [28]; and random errors in pad placement or its movement over the course of the study (around 1 cm). Considering these and the fact that light travels in a volume from source to detector, we could establish that the pad allowed good coverage of superior and middle temporal cortices, as well as inferior frontal gyrus, precentral gyrus, and the temporo-parietal junction. Fig. 5 shows an approximate depiction of the cortical coverages' estimation in both this study and the 4-month-old one reported by Minagawa in [11]. Blue circles indicate the approximate localization of channels in the 4-month-old study. Both studies used the international 10-20 method to place the optodes. In particular, T3 and T4 were used to attach the center optode in the lowest line, which corresponds to channel 6 in our present study. Taking into account this and the fact that the inter-optode separation in that study was 30 mm (cf. 25 mm in our present study), we can estimate the *relative* position of channels in that study using as reference the present channels' positions.

### Analyses

Intensity signal changes were converted into oxygenated hemoglobin (oxyHb) and deoxygenated (deoxyHb) hemoglobin concentration changes using the modified Beer-Lambert Law. We note that we use arbitrary units for expressing these estimated changes in concentration. Regions of artifact were then detected, in order to exclude these datapoints in the general linear model (GLM) analyses. A first GLM analysis ascertained that the standard hemodynamic response function model could be applied to these data. Subsequent GLMs proceeded to assess changes in concentration during specific stimulation types. Both GLM included nuisance parameters, in addition to those designed to capture responses to stimulation. Each of these aspects of data analysis is explained in more detail below.

#### Artifact detection

Movement artifacts cannot affect individual channels without affecting the actual optodes that establish the channels (if an infant's movement causes poor attachment, this physical event will affect a source or detector, and as a consequence all of the channels associated to it; see for example, [22]). Artifacts likewise affect both wavelengths, and it has been estimated that changes in concentration of total hemoglobin (oxyHb + deoxyHb) that are larger than 0.15 mM.mm in 100 ms are due to artifacts (e.g., [16]). We therefore adopt the exact same analyses and parameters in our own previous newborn work [22], as follows. First, total hemoglobin was band-pass filtered between 0.02 and 0.7 Hz and averaged over all of the shallow channels relevant for a given probe. A stretch of signal was labeled as artifacted if 2 successive samples (separated by 100 ms because of the 10 Hz sampling rate) had a jump in total hemoglobin concentration of more than 0.15 mM.mm. Once the average time series for each probe were labeled in such a way, we used them to create an artifact mask for each channel for both oxy and deoxy signals. For a given channel, a point in time was labeled as good data if neither the source nor the detector had an artifact at that point. Finally, if there were less than 20 s between two regions of artifact, the intervening signal was esteemed to be too short for a proper analysis and was also coded as artifacted.

On average, 16% of the time samples were excluded by a research assistant prior to analysis because they occurred before the experiment started or after it had finished; a further 8% were automatically declared as artifacted based on the criterion noted above; and about 1% of the time samples were masked because they were between two regions of artifact separated by less than 20 s. After these exclusions, the average number of trials per condition per channel and neonate was about 7.6 (SD  =  3.5). We have not found truly comparable information in previous fNIRS studies to allow us to assess whether our data was more or less artifacted than those of others. We inspected DBIfNIRS [14] to assess exclusion rates in previous infant fNIRS studies on healthy newborns. There were 26 such experiments, of which 15 did not report data exclusion. Among the remaining 11, 3 stated the newborns were awake (in quiet rest); whereas the others reported newborns were sleeping and/or quietly resting (i.e., sleeping status was not tracked carefully), as in our study. This comparison revealed that our data exclusion was comparable to previous work. The average proportion of infants excluded was 21%. Similarly here, taking into account that we require a minimum of 4 trials-worth of data, 19% of infant*channel*condition combinations (1062 over 40 newborns * 28 channels * 5 conditions) were excluded.

#### Nuisance regressors

Boxcar regressors were used to model baseline changes following artifacted regions. Sine and cosine regressors with T  =  2,3,…, n minutes, up to the duration of the session, were incorporated to account for slow nonlinear trends. The ‘artifacted’ stretches of the signal were excluded by giving them a weight of zero in the regression.

#### Hemodynamic Response Function (HRF) reconstruction

The HRF was reconstructed using GLM (as in much previous infant fNIRS work, e.g., [11], [13], [29]; see also [17] for a different use of GLM). To estimate the HRF, a linear model was fitted with 20 one-second boxcar regressors time-shifted by 0,1,… 19 seconds respectively from stimulus onset, collapsing across the experimental conditions within each infant and channel. These reconstructed HRFs were subsequently averaged across all channels and infants, for each depth separately. They were then compared to an adult HRF model shifted by delays at.1s intervals, within the range of (−2,8) seconds (i.e., first shifted by −2 seconds, then 1.9 seconds, etc.) The optimal phase was estimated as the delay that yielded the best regression coefficient. Finally, bootstrap resampling of the individual subjects' data [30] was used to generate 95% confidence intervals for this optimal phase (N  =  10,000).

As shown in Fig. 3, newborns' concentration changes in shallow channels correlated well with an unshifted version of the standard adult HRF model for oxyHb [R  = .96, mean phase 0.8s (95% CI −1,3)], but was poorer for deoxyHb [R  = .86, mean phase −0.7s (−2,8)], and for both signals in deep channels [oxyHb R  = .89, mean phase 2.2s (−1,3); deoxyHb R  = .53, mean phase 0.1s (−2,8)]. The observation that oxyHb is somewhat more reliable than deoxyHb has been heavily replicated in infant research (see discussions in [13], [31]). In view of this previous work and our own HRF fit, we concentrate subsequent analyses on oxyHb only and use the standard HRF model. Given that all the confidence intervals for the phase included zero, and that the mean phase for oxyHb in shallow channels was close to zero, no shift was imposed on the standard HRF used for subsequent GLMs.

#### Data analyses

The standard HRF model was convolved with the relevant stimulation paradigm, and the fit of this time series with the data was assessed for each channel and infant separately. Average *β* values were tested against zero using a t test across participants. We report all channels that were activated in each relevant condition at p ≤.05 uncorrected, but we considered a channel as indicating a significant effect only when the p from bootstrap resampling was ≤.05. For multiple-comparison correction, we used a single-step permutation test procedure, whereby the distribution of the the t-max statistics (i.e. the distribution of the maximum t-test statistics over all of the channels) under the null hypothesis was generated through random permutation of the condition labels across participants (see [32] for a detailed explanation of the procedure, and the multtest R package [33] for implementation).

Following some previous infant fNIRS work [13], we conducted a first analysis focused on channels that responded to the auditory stimulation. A GLM was fit contrasting all stimulation conditions versus silence, which should reveal channels responding to sound. Then, a *β* from a second, condition-based GLM corresponding to such activated channels, together with the *β* from a homologous channel in the opposite hemisphere, were submitted to a repeated measures Analysis of Variance (ANOVA), declaring Hemisphere (left, right) and Condition (macaque calls, human emotional vocalizations, L1, L2, and auditory control) as within-participant factors.

Additionally, we followed the adult study with similar stimuli by Joly and colleagues [12], and carried out a second analysis where stimulation was separated by condition, and a third in which specific contrasts across conditions were declared.
